# Clinical and genetic analyses of patients with lateralized overgrowth

**DOI:** 10.1186/s12920-022-01362-1

**Published:** 2022-09-30

**Authors:** Yoon-Myung Kim, Yena Lee, Yunha Choi, In Hee Choi, Sun Hee Heo, Jung Min Choi, Hyo-Sang Do, Ja-Hyun Jang, Mi-Sun Yum, Han-Wook Yoo, Beom Hee Lee

**Affiliations:** 1 Department of Pediatrics, Gangneung Asan Hospital, College of Medicine, University of Ulsan, Gangneung, South Korea; 2 Department of Pediatrics, Asan Medical Center Children’s Hospital, College of Medicine, University of Ulsan, Seoul, South Korea; 3 Asan Medical Center, Asan Institute for Life Sciences, College of Medicine, University of Ulsan, Seoul, South Korea; 4 Department of Laboratory Medicine and Genetics, Samsung Medical Center, Sungkyunkwan University School of Medicine, Seoul, South Korea; 5 Medical Genetics Center, Asan Medical Center, College of Medicine, University of Ulsan, Seoul, South Korea

**Keywords:** PIK3CA-related segmental overgrowth syndrome, Lateralized overgrowth, Alpelisib, Targeted exome sequencing

## Abstract

**Background:**

The genetic features and treatment strategies of lateralized overgrowth have been elusive. We performed this study to analyze the genetic characteristics and treatment results of propranolol- or alpelisib-treated patients with lateralized overgrowth.

**Methods:**

Fifteen patients with lateralized overgrowth were involved. Clinical characteristics and whole-body magnetic resonance imaging (WB-MRI) findings were evaluated. Targeted exome sequencing with a gene panel of affected tissue and peripheral white blood cells was performed. Propranolol was administered and treatment results were evaluated. The PIK3CA inhibitor alpelisib was prescribed via a managed access program.

**Results:**

The identified mutations were *PIK3CA* (n = 7), *KRAS* (n = 2), *PTEN* (n = 1), *MAP2K3* (n = 1), *GNAQ* (n = 1), *TBC1D4* (n = 1), and *TEK* (n = 1). Propranolol was prescribed in 12 patients, and 7 experienced mild improvement of symptoms. Alpelisib was prescribed in two patients with a *PIK3CA* mutation, and the reduction of proliferated masses after 1 year of treatment was proved by WB-MRI.

**Conclusions:**

Targeted exome sequencing identified various genetic features of lateralized overgrowth. Propranolol could be applied as an adjuvant therapy for reducing vascular symptoms, but a PIK3CA inhibitor would be the primary therapeutic strategy for PIK3CA-related overgrowth syndrome.

**Supplementary Information:**

The online version contains supplementary material available at 10.1186/s12920-022-01362-1.

## Introduction

Overgrowth syndrome (OS) refers to a group of disorders with abnormal, excessive tissue proliferation, which can be classified as general or segmental. Segmental overgrowth, or lateralized overgrowth, includes the phosphoinositide-3-kinase, catalytic, alpha polypeptide (PIK3CA) − related overgrowth spectrum (PROS), mosaic RASopathies, PTEN hamartoma tumor syndrome and Beckwith–Wiedemann spectrum [1, 2]. The abnormal proliferation of various types of tissues such as vascular, musculoskeletal or adipose are observed in these disorders which is caused by somatic mosaicism [2].

The PI3K/Protein Kinase B (AKT)/mechanistic target of rapamycin (mTOR) signaling pathway has major roles in cell growth, proliferation, and differentiation [3]. The Ras family of small GTPase proteins (RAS)/mitogen-activated protein kinases (MAPK) pathway also interacts with the PI3K/AKT/mTOR pathway. The phosphatase and tensin homolog (PTEN) and tuberous sclerosis complex (TSC) 1 and 2 are the negative regulators of PI3K and mTOR, respectively [3].

PROS account for Klippel–Trenaunay syndrome (KTS), Megalencephaly-Capillary Malformation syndrome (M-CM), congenital lipomatous lateralized overgrowth of the trunk, lymphatic, capillary, venous, and combined-type vascular malformations, epidermal nevi, skeletal, and spinal anomalies (CLOVES) syndrome [3]. Additional OSs such as Proteus syndrome is related to the activation of the PI3K-AKT-mTOR pathway [4].

There is an ongoing effort to treat OS using inhibitors of PI3K, AKT, or mTOR [3], and a PIK3CA inhibitor named alpelisib (Novartis Pharmaceuticals Corporation) was proposed to show efficacy in patients with PROS/CLOVES[5, 6]. This drug was under investigation in clinical trials [7] and the Retrospective Chart Review Study of Patients With PIK3CA-Related Overgrowth Spectrum Who Have Received Alpelisib (EPIK-P1) has been completed [8]. The phase II study (EPIK-P2) [9] is on process and alpelisib has been recently approved by the United States Food and Drug Administration (FDA) for adult and pediatric patients two years of age and older with severe manifestations of PROS [10].

Propranolol, a beta-blocker, has been widely used for the treatment of infantile hemangiomas, and several previous studies have suggested that propranolol might negatively regulate the AKT/mTOR pathway [11–13]. The most recent study identified that propranolol inhibits the transcription factors of sex-determining region Y (SRY) box transcription factor 18 (SOX18) which plays and important role in endothelial cell differentiation during blood vessel development and angiogenesis [14]. Indeed, propranolol has shown partial efficacy in the regression of vascular masses in patients with KTS [15, 16]. However, the effects of propranolol are limited to vascular malformation with no impact on non-vascular growth [14].

In the present study, the genetic analysis, including PIK3CA and its related pathways, of patients with lateralized overgrowth were explored. In addition, we report the experiences of long-term propranolol treatment in segmental OS and of alpelisib in cases with somatic *PIK3CA* mutation.

## Materials and methods

### Subjects and evaluation of clinical characteristics

This study was a single-center, open-label, non-randomized, prospective observational study performed at the Asan Medical Center, Seoul, Korea, from February 2014 to May 2020. The study was approved by the Institutional Review Board of Asan Medical Center (no. 2020–1628). Written informed consent was obtained from all participants and/or their legal guardians and all methods were performed in accordance with the relevant guidelines and regulations. Fifteen patients with clinical features of lateralized overgrowth who attended Asan Medical Center during the study period were enrolled. The affected areas were evaluated by physical examination and whole-body magnetic resonance imaging (WB-MRI). Evidence of cutaneous capillary malformation, such as port-wine stain, telangiectasia and angiokeratoma were evaluated by physical examination. The affected area was classified into vascular malformation, musculoskeletal overgrowth and adipose tissue proliferation. WB-MRI was performed yearly after treatment in all patients to evaluate the change of the affected limb. Medical photographs were taken yearly.

### Genetic analysis

Genetic analysis was performed using a customized gene panel. Exome sequencing was performed using genomic DNA extracted from the affected tissue and peripheral blood leukocytes. The affected tissue was obtained by skin biopsy from the regions with hypertrophy or cutaneous capillary malformation. Exomes were captured using a Celemics Custom Panel (Celemics Inc., Seoul, Korea), which enriches a 372,068 bp region spanning 143 genes related to cell signaling pathways (Additional File 1: Table S1). The list of the targeted genes is presented in the Additional File 1. Sequencing was performed on the NextSeq platform (Illumina Inc., San Diego, CA, US). The mean depth of coverage was 878 reads per base with a 30X coverage of 99.3% for the affected tissue-extracted DNA sequencing. The mean depth of coverage was 346 reads per base with a 30X coverage of 99.0% for the blood-extracted DNA sequencing. Sequence reads were aligned to the reference genome, hg19, using Burrows–Wheeler Aligner (version 0.7.12, MEM algorithm) [17]. Duplicate reads were removed using Picard tools version 1.96. The Genome Analysis Toolkit (GATK version 3.7) was used for local realignment and base quality recalibration. Variant calling was performed using GATK MuTect2 and HaplotypeCaller [18] for tissue and blood, respectively. Common variants with minor allele frequency ≥ 1% were filtered out using public databases such as the Genome Aggregation Database, Exome Variant Server, and 1000 Genomes Browser. Population-specific common variants were further filtered out using the Korean Reference Genome Database. Variants were annotated using Variant Effect Predictor 88 and Oncotator (version 1.9.2). Candidate variants were manually curated using Integrated Genome Viewer.

### Treatment strategies

The off-label use of propranolol was suggested in all the fifteen patients, and 12 of them agreed with the treatment. Treatment with propranolol was initiated at a dose of 0.5 mg/kg/day, which was subsequently increased to a maximum dose of 4 mg/kg/day. WB-MRI was performed before treatment and one year after treatment to analyze the change in the volume of the affected extremities. Responses to the SF-36 version 2 short-form health survey questionnaires [19] were acquired before treatment and after one year of treatment to evaluate changes in quality of life. SF-36 questions yield eight subscales and two summaries of physical and mental component scores. The questionnaires was conducted only on five patients who were older than adolescence, because the SF-36 survey is not suitable for children. Four out of 5 completed the pre- and post-treatment survey.

Alpelisib was administered in two patients for clinical trial via a managed access program (MAP) approved by Novartis Pharmaceuticals Corporation (Novartis/CBYL719X2001I MAP ID 17,746/17751). The application of alpelisib was permitted only for two volunteers. Alpelisib was provided as a 50 mg or 200 mg coated tablet and administered orally once daily. A fixed dose of 250 mg was administered to an adult patient and 50 mg to a pediatric patient as previously described [5] and according to the manufacturer’s instruction. The volume change in the extremities was measured and compared using WB-MRI before treatment and after one year of treatment.

## Results

### Clinical presentation and diagnosis

**Fig. 1:**
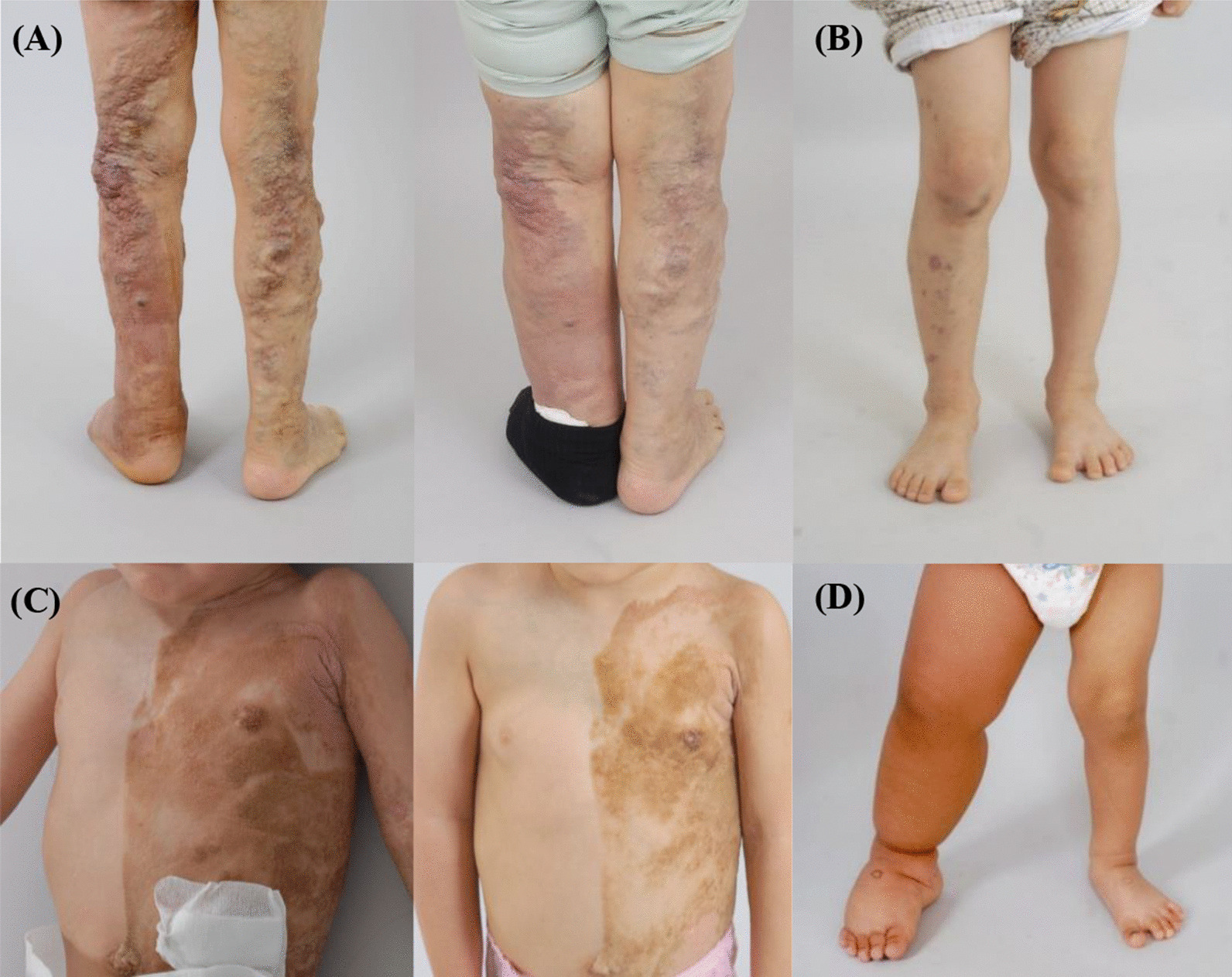
Clinical manifestations of patients. **A** Patient 5 with a *PIK3CA* mutation with prominent superficial venous engorgement. The superficial venous engorgement showed improvement after 3 years of propranolol administration. **B** Patient 1 with a *PIK3CA* mutation showing port-wine stain of the skin and hypertrophy of the right leg. **C** Patient 10 with a *PTEN* mutation presented with epidermal nevus. The color of the epidermal nevus faded after 6 months of propranolol administration. **D** Patient 9 with a *KRAS* mutation showing hypertrophy of the right leg

Fifteen patients (11 males and 4 females) with a mean age of 15.6 ± 19.3 years (range, 0.25–53 years) were enrolled. None of the patients had a family history of overgrowth. Eight patients (8/15, 53.3%) showed overgrowth of the lower extremities—five in the right, two in the left, and one in both. Seven patients (7/15, 46.7%) showed overgrowth of both the lower and upper extremities—three in the left, two in the right and two in both (Additional File 1: Fig. S1). Eight patients showed limb length discrepancy. Vascular malformation such as cutaneous capillary malformation or venous engorgement were identified in all patients (Table 1). Cutaneous capillary malformation presented as port-wine stain in 11 patients (11/15, 73.3%). Patient 10 had a pigmentary skin macules presenting as epidermal nevi on the left trunk and extremities (Fig. 1). Venous engorgement of extremities was identified in nine patients (9/15, 60%) using WB–MRI. Bone overgrowth or malformation were identified in four patients (4/15, 26.7%) (Table 1). No patients with adipose tissue proliferation were observed. Other clinical features included lymphatic malformation (n = 4, 26.6%), leg or spinal arteriovenous malformation (n = 2, 13.3%), hemimegalencephaly (n = 1, 6.7%), seizure (n = 1, 6.7%), and one-sided blindness (n = 1, 6.7%) (Table 1). More clinical photos of the patients are presented in the Additional File 1.

**Table 1:** Clinical characteristics of patients with lateralized overgrowth syndrome in the present study

Patient (Sex/age)	Clinical features				Genetic diagnosis (mutated gene)
	Lateralized overgrowth	Cutaneous capillary malformation	Venous engorgement	Others	
1 (M/7y)	Right leg	Right leg	+	–	PROS (*PIK3CA*)
2 (M/8y)	Left arm and leg	Trunk, both arms, left leg	–	Seizure, ipsilateral long 2nd toe	PROS (*PIK3CA*)
3 (F/39y)	Left arm and leg	Face, trunk, Left arm	+	–	PROS (*PIK3CA*)
4 (M/10 m)	Both legs	Left neck and shoulder	–	Hemimegalencephaly, Macrodactly of feet	PROS (*PIK3CA*)
5 (F/53y)	Both upper arms and legs	Both legs	+	Pulmonary thromboembolism	PROS (*PIK3CA*)
6 (F/4 m)	Left arm and leg	Trunk, left face, neck	–	–	PROS (*PIK3CA*)
7 (M/4 m)	Right arm and leg	Trunk, both arms and legs	+	–	PROS (*PIK3CA*)
8 (M/7y)	Right legs	None	+	–	Mosaic RASopathies (*KRAS*)
9 (M/2y)	Right legs	None	+	Lumbosacral AVM, Lymphatic malformation	Mosaic RASopathies (*KRAS*)
10 (F/2y)	Left arm and both legs	None	+	Epidermal nevi, Chest wall AVM, Lymphatic malformation	PTEN hamartoma tumor syndrome (*PTEN*)
11 (M/3y)	Left leg	None	+	Left tibial hypertrophy, lymphatic malformation	Mosaic RASopathies (*MAP2K3*)
12 (M/17y)	Right arm and leg	Face, neck, right side of trunk, right arm, both legs	+	Ipsilateral eye blindness, mental retardation	Sturge–Weber syndrome (*GNAQ*)
13 (M/47y)	Right leg	Right leg	–	lymphatic malformation	(*TBC1D4*)
14 (M/5y)	Left leg	Left leg	–	–	TEK-related vascular malformation (*TEK*)
15 (M/43y)	Right leg	Face, neck, trunk, left arm, both legs	–	Right hemifacial bone prominency	Not determined

PROS, PIK3CA-related overgrowth spectrum; KTS, Klippel-Trenaunay syndrome; AVM, arteriovenous malformation; M, male; F, female

### Genetic diagnosis

The somatic variant was considered significant if the variant was not observed in a public genome database, was predicted to alter protein function significantly according to multiple in silico analyses and the American College of Medical Genetics (ACMG) guidelines [20], and was then observed in the affected tissue with a variant allele frequency (VAF) > 0.01 (Tables 2 and 3). The VAF in the affected tissue was also compared to that in non-affected tissue (blood leukocytes) of each patient. Significant mutations were observed in the affected tissue of 14 patients (93.3%) with the exception for patient 15. The identical mutations were also identified in the blood of three patients, and their VAF values are shown in Table 2. Seven patients (46.7%) carried a *PIK3CA* mutation; two patients (13.3%) had a *KRAS* mutation. Other identified mutations included *PTEN* (n = 1, 6.7%), *MAP2K3* (n = 1, 6.7%), *GNAQ* (n = 1, 6.7%), *TBC1D4* (n = 1, 6.7%), and *TEK* (n = 1, 6.7%) (Table 2). According to the ACMG guidelines, seven variants were predicted as pathogenic, five variants as likely pathogenic, and two variants as “variant of unknown significance” (VUS). All of the identified mutations, except for *TEK*, can be found in the Catalogue of Somatic Mutations in Cancer (COSMIC) database [21]. The identified *PIK3CA* mutations have been previously reported in other patients with PROS [22, 23], and the *KRAS* and *TEK* mutations in patients with vascular malformation [24, 25], but the *MAP2K3* and *TBC1D4* mutations have not been previously reported.

**Table 2:** Genetic characteristics of identified mutations in patients

Patient	Mutated gene	DNA sequence	Amino acid change	VAF (tissue)	VAF (blood)	Depth (X) (tissue)	Depth (X) (blood)	COSMIC ID	Biopsy site and lesion
1	*PIK3CA*	c.1636C > A	p.Gln546Lys	0.048	0	513	594	COSM766	Right thigh, cutaneous capillary malformation
2	*PIK3CA*	c.2740G > A	p.Gly914Arg	0.182	0	765	326	COSM3205660	Left arm, cutaneous capillary malformation
3	*PIK3CA*	c.1345C > A	p.Pro449Thr	0.131	0	721	323	COSM18601	Left arm, cutaneous capillary malformation
4	*PIK3CA*	c.1633G > A	p.Glu545Lys	0.023	0	633	352	COSM763	Left leg, cutaneous capillary malformation
5	*PIK3CA*	c.1357G > A	p.Glu453Lys	0.127	0	1554	398	COSM12584	Left leg, cutaneous capillary malformation
6	*PIK3CA*	c.3073A > G	p.Thr1025Ala	0.081	0	1149	314	COSM771	Neck, hemangioma
7	*PIK3CA*	c.2908G > A	p.Glu970Lys	0.023	0.036	890	239	COSM94980	Lower leg, cutaneous capillary malformation
8	*KRAS*	c.35G > A	p.Gly12Asp	0.041	0	1080	212	COSM521	Right lower leg, skin of overgrowth lesion
9	*KRAS*	c.35G > A	p.Gly12Asp	0.058	0	571	391	COSM521	Right lower leg, skin of overgrowth lesion
10	*PTEN*	c.755A > T	p.Asp252Val	0.712	0.481	880	281	COSM3368151	Abdomen skin, epidermal nevus
11	*MAP2K3*	c.696 + 1G > A	p.(?)	0.042	0.088	796	341	COSM560209	Left inguinal area, skin of overgrowth lesion
12	*GNAQ*	c.548G > A	p.Arg183Gln	0.039	0	1038	248	COSM52975	Right flank area, nevus flammeus
13	*TBC1D4*	c.667G > A	p.Asp223Asn	0.026	0	952	273	COSM6797461	Right lower leg, skin of overgrowth lesion
14	*TEK*	c.3324_3334del	p.Glu1109Leufs Ter5	0.046	0	544	567	–	Left buttock, skin of overgrowth lesion
15	Not found		–	–	–	1074	322	–	Abdomen, cutaneous capillary malformation

VAF, variant allele frequency; COSMIC, Catalogue of Somatic Mutations in Cancer

**Table 3:** In silico identification of mutations in patients

Patient	Mutated gene	DNA sequence change	SIFT	Mutation taster	LRT	PROVEAN	CADD phred score	Predicted pathogenicity^*^
1	*PIK3CA*	c.1636C > A	Tolerated	Disease causing	Deleterious	Neutral	25.2	Pathogenic
2	*PIK3CA*	c.2740G > A	Deleterious	Disease causing	Deleterious	Deleterious	31	Pathogenic
3	*PIK3CA*	c.1345C > A	Deleterious	Disease causing	Deleterious	Deleterious	28.4	Likely pathogenic
4	*PIK3CA*	c.1633G > A	Deleterious	Disease causing	Deleterious	Deleterious	33	Pathogenic
5	*PIK3CA*	c.1357G > A	Tolerated	Disease causing	Deleterious	Neutral	32	Likely pathogenic
6	*PIK3CA*	c.3073A > G	Tolerated	Disease causing	Deleterious	Deleterious	23.2	Likely pathogenic
7	*PIK3CA*	c.2908G > A	Tolerated	Disease causing	Neutral	Neutral	22.8	Likely pathogenic
8	*KRAS*	c.35G > A	Deleterious	Disease causing	Deleterious	Deleterious	25.3	Pathogenic
9	*KRAS*	c.35G > A	Deleterious	Disease causing	Deleterious	Deleterious	25.3	Pathogenic
10	*PTEN*	c.755A > T	Deleterious	Disease causing	Deleterious	Deleterious	27.3	Likely pathogenic
11	*MAP2K3*	c.696 + 1G > A	–	Disease causing	–	–	26.8	VUS
12	*GNAQ*	c.548G > A	Deleterious	Disease causing	Deleterious	Deleterious	35	Pathogenic
13	*TBC1D4*	c.667G > A	Deleterious	Disease causing	Deleterious	Deleterious	34	VUS
14	*TEK*	c.3324_3334del	–		–	–	–	Pathogenic

SIFT, Sorting Intolerant For Tolerant; LRT, Likelihood Ratio Test; PROVEAN, Protein Variation Effect Analyzer; CADD, Combined Annotation Dependent Depletion; VUS, variant of unknown significance;*, As presented in the consensus statement of the ACMG (American College of Medical Genetics)

Among the 15 patients with lateralized overgrowth, the genetic diagnosis was PROS in 7 patients, mosaic RASopathies in 3 patients, PTEN hamartoma tumor syndrome in 1 patient, and Sturge–Weber syndrome in 1 patient (Table 1). Further genetic evaluation of patient 15 was not available.

### Treatment outcomes

Propranolol treatment was proposed to all patients and 12 patients finally received it (Table 3). The total duration of treatment was 26 ± 13.7 months (range, 12–50 months). The initial starting dose of propranolol was 0.76 ± 0.29 mg/kg/day (range, 0.4–1.3 mg/kg/day), and the escalated maximum dose, decided based on the patients’ tolerance, was 3.2 ± 1.1 mg/kg/day (range, 0.5–4.0 mg/kg/day).

Seven patients experienced mild improvement of symptoms: relief of pain, extended range of motion, and a mild decrease in cutaneous swelling and capillary lesions (Table 4 and Fig. 1). The responses to the SF-36 version 2 of short-form health survey questionnaires were available in four patients. The mean physical component score of SF-36 changed from 62.3 ± 18.9 to 63.75 ± 24.8 after propranolol treatment (Wilcoxon signed-rank test, *p* = 1.0). The mental component score also changed from 59.7 ± 11.6 to 60.6 ± 17.4 (Wilcoxon signed-rank test, *p* = 1.0). Patient 5 showed improvement in the SF-36 score after treatment, but there was no significant difference in the remaining patients (Table 4). The WB-MRI images after treatment were compared, but none of the patients showed obvious decrease in the total volume of the affected extremities (Table 4). Patients 5 and 11 showed aggravation of leg swelling after discontinuing propranolol, but they regained the treatment effect again after reintroducing propranolol. Patient 5 showed improvement of cutaneous vascular symptoms with a 3-year administration of propranolol (Fig. 1). Three patients experienced transient dizziness or bradycardia, but they were able to continue treatment at a lower dose without further side effects.

**Table 4:** Clinical profiles of patients receiving propranolol treatment

Patient	Maximum dose (mg/kg/day)	Duration (month)	Adverse event	Clinical improvement	Change in follow up WB-MRI findings	SF-36 physical score		SF-36 mental score	
						Before treatment	After treatment	Before treatment	After treatment
1	3	18	Dizziness	None	No change	–	–	–	–
2	1.8	12	None	None	No change	–	–	–	–
3	2.4	12	Bradycardia, chest pain	Relieved leg pain	Less prominent superficial veins of leg	61.6	50.6	67.8	53.4
5	3.3	44	None	Increased upper arm range of motion, decreased varicosities and leg swelling, improvement in ambulation	No change	79.4	91.3	60.6	73.8
6	4	21	None	Slight improvement of left arm and neck hypertrophy	Less prominent superficial veins of neck	–	–	–	–
7	4	41	None	None	No change	–	–	–	–
8	4	50	None	None	No change	–	–	–	–
10	3.6	21	Dizziness	Improved skin pigmentation	No change	–	–	–	–
11	4	39	None	Decreased leg swelling	No change	–	–	–	–
12	4	25	None	Decreased skin pigmentation	No change	72.2	73.8	67.3	65.93
13	0.5	12	None	Decreased foot swelling	No change	91.9	–	90.7	–
15	4	17	None	Decreased back pain, increased shoulder range of motion, decreased skin pigmentation of thigh	No change	36.3	33.1	42.9	35
Average	3.2 ± 1.1	26 ± 13.7				62.3 ± 18.9	63.75 ± 24.8	59.7 ± 11.6	60.6 ± 17.4
*p*-value						1.0		1.0	

WB-MRI, Whole Body Magnetic Resonance Imaging; SF-36, SF-36 version 2 of short-form health survey questionnaires

**Fig. 2:**
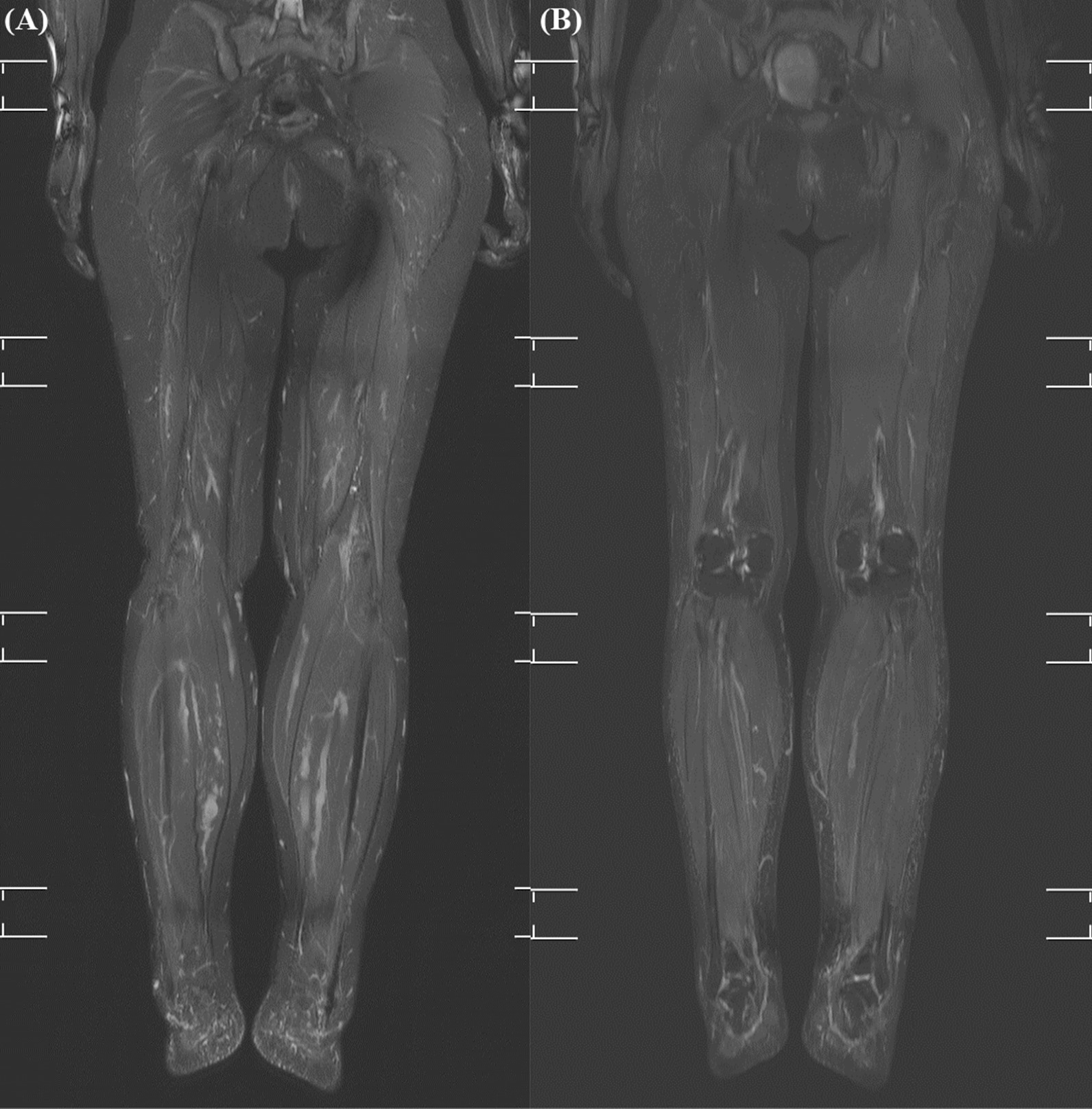
Change in the MRI findings of patient 3 after the administration of alpelisib. **A** The MRI image of both lower legs before treatment **B** The MRI image of both legs after treatment. The extent of fine stranding of the subcutaneous layer of the bilateral distal lower legs decreased. Minimal improvement of the tortuous and dilated deep and superficial venous structure in the lower extremities was observed

Alpelisib was administered for 18 months in patients 2 and 3, who had a somatic *PIK3CA* mutation and hypertrophy of the extremities. Propranolol administration was discontinued and WB-MRI was performed before the initiation of alpelisib treatment. There were no adverse events during the study period and drug administration was continued. Patient 2 had a lateralized overgrowth of the left leg and was 10 years old at the start of the clinical trial (50 mg daily). In patient 2 who was a growing child, the volumes of the left and right leg before treatment were 8351.6 cm^3^ and 7758.6 cm^3^, respectively. After 1 year of treatment with alpelisib, the volumes of the left and right leg increased to 9013.2 cm^3^ and 8542.2 cm^3^, respectively. The volume increase rate of the left and right leg were 7.9% and 10.1%, respectively. There was a 7.6% difference between the volumes of both legs before treatment, but this difference slightly decreased to 5.5% after 1 year of treatment. Patient 3 had hypertrophy of both lower legs and was 42 years old at the start of the clinical trial (250 mg daily). In patient 3, the volumes of the left and right leg before treatment were 19,867.3 cm^3^ and 18,239.1 cm^3^, respectively. After 1 year of treatment with alpelisib the volumes of the left and right leg decreased to 18,000.9 cm^3^ and 16,570.3 cm^3^, respectively. The volume decrease rates of the left and right leg were 9.4% and 9.1%, respectively (Fig. 2).

## Discussion

The current study described the molecular genetic analysis of patients with lateralized overgrowth. By utilizing deep sequencing of the genes related to the PIK3CA/AKT/mTOR and RAS/MAPK pathways, we were able to identify the known genetic variants of this condition. Moreover, by comparing the VAF in the affected tissues to that in the normal tissues, we could detect mutations with low levels of mosaicism.

Most of our patients were identified as having PROS, but *KRAS*, *PTEN*, *MAP2K3*, *GNAQ*, *TBC1D4*, and *TEK* mutations were also identified. Careful physical examination of dysmorphic features can help to identify a recognizable pattern in some cases, but differential diagnosis is challenging in other cases because of the variety of overlapping phenotypes between the disorders with lateralized overgrowth. The degree of mosaicism varies widely from individual to individual, and the diverse phenotypes also makes it difficult to diagnose. In these cases, genome testing can enable differential diagnosis. To improve the genetic detection rate, the affected tissue, such as vascular malformation tissue, should be obtained. In addition, as the mutation exists in a mosaic pattern, deep sequencing using massively parallel sequencing techniques needs to be applied. Therefore, targeted gene panel testing with deep sequencing is useful for the genetic diagnosis of lateralized overgrowth. The overall molecular diagnostic yield of OS has been reported as up to 45% in the affected tissues [26]. In the present study, a customized panel was used to sequence as many genes as possible that are related to the PIK3CA/AKT/mTOR and RAS/MAPK pathways (the mean depth of coverage was 878 reads per base) and thereby enhance the overall genetic detection rate. Consequently, we were able to identify genetic defects in most of our patients. Some cases of isolated lateralized overgrowth are caused by methylation defects on the region of chromosome 11p15 [2], so methylation studies could be considered if the causative gene is not identified by exome sequencing.

The levels of mosaicism can be as low as < 5% in the affected tissues of patients with PROS [27]. In the present study, additional deep-sequencing tests with blood samples were helpful in diagnosing seven patients with low levels of mosaicism. Germline filtering also helps to identify germline mutations, as in patient 10 of our study [28].

The association between the genetic variants and overgrowth in the patients in the present study is supported by multiple observations. All the variants found in the present study have not been reported in the normal population but, importantly, have been identified in the affected tissues of the enrolled patients. Furthermore, using multiple in silico prediction tools and per the ACMG guidelines, these variants are predicted to alter the protein function [20]. Besides PIK3CA, variants related to the RAS/MAPK pathway were identified in *KRAS* and *MAP2K3* mutations in three patients. Mosaic RASopathy variants are related to vascular malformation [24]. The *MAP2K3* variant found in our study has not been reported, but considering the cell proliferation function and correlation with tumorigenesis of MAP2K3 [29], it could have a relation with overgrowth. The germline *PTEN* mutation (in patient 10) causes epidermal nevus and mild vascular malformation of the buttock and thigh that is associated with PTEN hamartoma tumor syndrome. The high VAF of 0.7 from the blood sample in this patient might have resulted from partial deletion or second hit of the wild-type *PTEN* allele [30]. The *GNAQ* mutation in patient 12 explains the capillary malformation involving OS observed in Sturge–Weber syndrome [31–33]. The *TEK* mutation in patient 13 can cause multiple sporadic venous malformations [25, 34, 35]. Although the association between the *TBC1D4* mutation and lateralized overgrowth in patient 14 is not clear, the same somatic mutation has been reported in large intestinal adenocarcinoma tissue [36]. TBC1D4 is a GTPase-activating protein that functions downstream of AKT and seems to regulate the proliferation of multiple cell types [37–39].

Although no significant improvement was observed in the affected areas following propranolol treatment, some patients experienced improvement of pain, range of motion, cutaneous symptoms, and quality of life. Notably, rapid worsening of symptoms was observed in some patients after discontinuation of propranolol. Propranolol may interupt angiogenesis[14] and subtly reduce the burdens caused by vascular malformations. Propranolol is a drug with fewer serious side effects; therefore, it could be used as an adjunct to relieve vascular symptoms in patients with lateralized overgrowth.

The mTOR inhibitor sirolimus has been clinically administered at low doses and showed a modest reduction in OS symptoms. However, 72% of participants had at least one adverse event; therefore, risk–benefit evaluations must be carefully considered when deciding on a treatment regimen for these patients[40]. The first evidence for the use of alpelisib in patients with PROS showed promising efficacy and no substantial side effects [5]. There was an improvement in vascular tumor size, congestive heart failure, lateralized overgrowth and scoliosis [5]. The two patients with PROS in the present study who participated in the alpelisib MAP [7] experienced a reduction in the degree of hypertrophy after administration for 1 year. There were no side effects in these patients following an 18-months trial of alpelisib. A low-dose alpelisib of 25 mg once daily therapy administered to two infants showed efficacy with no adverse events in the recent study [41]. Alpelisib has been approved for pediatric patients two years of age and older with a single dose of 50 mg once daily. Nonetheless, some common side effects of alpelisib include hyperglycemia, diarrhea, nausea, fatigue, stomatitis, and pneumonitis, and patients should therefore be carefully monitored [42]. PI3K/AKT/mTOR inhibitors would also affect the metabolism of healthy cells [3], and the need for life-long therapy with these inhibitors raises concerns of unknown side effects. Further research on the treatment of patients with PROS with alpelisib is required to dispel the concerns with dosage and adverse events.

Several limitations should be addressed in the present study. Although most of the identified mutations have also been found in vascular malformation tissues of other studies, more evidence with the *MAP2K3* and *TBC1D4* variants is needed to prove their association with overgrowth. The low levels of mosaicism in some patients means that the causality of the mutation remains elusive. The alleles with mosaic mutations could be present in only a subset of cells, and the causative variant can easily be missed without a precise technical procedure. If the depth of sequencing was higher, we might have detected more potential mosaic variants in this study. Achieving a much higher reading depth is needed to increase the low frequency fraction variant detection sensitivity of mosaicism and avoid misinterpretation [43, 44]. As an open-label, non-randomized, observational study, the objective evaluation of the efficacy of propranolol was impossible.

In conclusion, customized panel-gene deep-sequencing enhanced the genetic diagnosis in patients with lateralized overgrowth syndrome, which furthermore identified the potentially causative new variants in *MAP2K3* and *TBC1D4*. Propranolol could be used as an adjuvant therapy for decreasing vascular symptoms in lateralized overgrowth patients. Targeted therapy considering genetic causes would be the leading therapeutic strategy of overgrowth syndrome in the future.

## Supplementary Information

**Additional file 1. **Supplementary table and figure.

## Data Availability

All data supporting the presented results are included in this published article. The raw data of whole-exome sequencing of the patient in this study are not publicly available to protect participant confidentiality, but they are available from the corresponding author on reasonable request. Please contact Professor BH Lee at the Department of Medical Genetics in the Asan Medical Center Children’s hospital for any requests to access the data. Reference sequences for *PIK3CA* (NC_000003.12), *KRAS* (NC_000012.12), *PTEN* (NC_000010.11), *MAP2K3* (NC_000017.11), *GNAQ* (NC_000009.12)*, TBC1D4* (NC_000013.11) and TEK (NC_000009.12) are available in the GenBank repository. The links to the GenBank repositories are as follows; *PIK3CA*(https://www.ncbi.nlm.nih.gov/nuccore/NC_000003.12?from=179148126&to=179240093&report=genbank), *KRAS*(https://www.ncbi.nlm.nih.gov/nuccore/NC_000012.12?from=25205246&to=25250929&report=genbank&strand=true), *PTEN*(https://www.ncbi.nlm.nih.gov/nuccore/NC_000010.11?from=87863625&to=87971930&report=genbank), *MAP2K3*(https://www.ncbi.nlm.nih.gov/nuccore/NC_000017.11?from=21284711&to=21315240&report=genbank), *GNAQ*(https://www.ncbi.nlm.nih.gov/nuccore/NC_000009.12?from=77716097&to=78031811&report=genbank&strand=true), *TBC1D4*(https://www.ncbi.nlm.nih.gov/nuccore/NC_000013.11?from=75283503&to=75482169&report=genbank&strand=true), and *TEK*(https://www.ncbi.nlm.nih.gov/nuccore/NC_000009.12?from=27109141&to=27230178&report=genbank).
