# Supplementary material for: Clinical and genetic analyses of patients with lateralized overgrowth
Source: BMC Med Genomics. 2022 Sep 30;15:206. doi: 10.1186/s12920-022-01362-1 (PMC9524090; doi:10.1186/s12920-022-01362-1)
Supplement: Supplementary file 1 — Additional file 1. Supplementary table and figure. [file 12920_2022_1362_MOESM1_ESM.docx]

Additional File 1: Table S1. List of the targeted genes.

| No. | Gene | Number of target region | Size of target region (bp) |
| --- | --- | --- | --- |
| 1 | *PIK3CD* | 22 | 4,013 |
| 2 | *MTOR* | 58 | 9,865 |
| 3 | *SDHB* | 8 | 1,151 |
| 4 | *PIK3R3* | 11 | 1,877 |
| 5 | *PRKAA2* | 9 | 2,019 |
| 6 | *JAK1* | 24 | 4,425 |
| 7 | *GLMN* | 18 | 2,437 |
| 8 | *NRAS* | 4 | 730 |
| 9 | *THEM4* | 6 | 963 |
| 10 | *SHC1* | 12 | 2,235 |
| 11 | *LMNA* | 15 | 2,819 |
| 12 | *GLUL* | 6 | 1,362 |
| 13 | *MAPKAPK2* | 12 | 1,591 |
| 14 | *PFKFB2* | 15 | 2,184 |
| 15 | *FH* | 10 | 1,851 |
| 16 | *AKT3* | 14 | 2,044 |
| 17 | *LPIN1* | 24 | 3,975 |
| 18 | *KCNK3* | 2 | 1,248 |
| 19 | *SOS1* | 23 | 4,896 |
| 20 | *ANTXR1* | 21 | 2,304 |
| 21 | *BCL2L11* | 9 | 1,097 |
| 22 | *ACVR1* | 9 | 1,890 |
| 23 | *PDK1* | 13 | 1,769 |
| 24 | *BMPR2* | 13 | 3,637 |
| 25 | *PIKFYVE* | 41 | 7,921 |
| 26 | *IRS1* | 3 | 3,725 |
| 27 | *EIF4E2* | 10 | 1,198 |
| 28 | *VHL* | 5 | 951 |
| 29 | *RAF1* | 17 | 2,669 |
| 30 | *MAPKAPK3* | 10 | 1,549 |
| 31 | *GSK3B* | 12 | 1,773 |
| 32 | *PIK3CB* | 22 | 4,059 |
| 33 | *PDCD10* | 7 | 903 |
| 34 | *PIK3CA* | 20 | 3,979 |
| 35 | *KDR* | 30 | 5,258 |
| 36 | *FAT4* | 18 | 15,485 |
| 37 | *GAB1* | 11 | 2,615 |
| 38 | *VEGFC* | 8 | 1,512 |
| 39 | *RICTOR* | 39 | 6,733 |
| 40 | *PRKAA1* | 11 | 2,205 |
| 41 | *PIK3R1* | 17 | 2,977 |
| 42 | *AGGF1* | 15 | 2,670 |
| 43 | *RASA1* | 27 | 4,092 |
| 44 | *PPP2CA* | 7 | 1,210 |
| 45 | *FLT4* | 30 | 5,276 |
| 46 | *ATXN1* | 3 | 2,437 |
| 47 | *MAPK14* | 13 | 1,723 |
| 48 | *CDKN1A* | 3 | 708 |
| 49 | *VEGFA* | 10 | 1,538 |
| 50 | *SGK1* | 17 | 2,600 |
| 51 | *CCM2* | 11 | 1,868 |
| 52 | *HSPB1* | 3 | 738 |
| 53 | *YWHAG* | 2 | 824 |
| 54 | *KRIT1* | 16 | 2,851 |
| 55 | *SND1* | 24 | 3,693 |
| 56 | *RHEB* | 8 | 875 |
| 57 | *PPP2CB* | 7 | 1,210 |
| 58 | *IKBKB* | 22 | 3,244 |
| 59 | *LYN* | 12 | 2,019 |
| 60 | *DEPTOR* | 10 | 1,507 |
| 61 | *PTK2* | 36 | 4,900 |
| 62 | *RPS6* | 6 | 990 |
| 63 | *TEK* | 23 | 4,287 |
| 64 | *GNAQ* | 7 | 1,360 |
| 65 | *SYK* | 13 | 2,428 |
| 66 | *MAPKAP1* | 12 | 2,063 |
| 67 | *ENG* | 15 | 2,560 |
| 68 | *PTPA* | 11 | 1,424 |
| 69 | *TSC1* | 22 | 4,315 |
| 70 | *MAP3K8* | 7 | 1,677 |
| 71 | *GDF2* | 2 | 1,370 |
| 72 | *DDIT4* | 2 | 779 |
| 73 | *PTEN* | 11 | 1,999 |
| 74 | *PIK3AP1* | 17 | 3,098 |
| 75 | *CHUK* | 22 | 3,041 |
| 76 | *PDCD4* | 12 | 1,876 |
| 77 | *HRAS* | 5 | 852 |
| 78 | *ILK* | 12 | 1,839 |
| 79 | *WEE1* | 16 | 2,095 |
| 80 | *RAG1* | 1 | 3,172 |
| 81 | *ATG13* | 17 | 2,312 |
| 82 | *VEGFB* | 7 | 931 |
| 83 | *BAD* | 3 | 627 |
| 84 | *CCND1* | 6 | 1,048 |
| 85 | *GAB2* | 10 | 2,417 |
| 86 | *SDHD* | 4 | 640 |
| 87 | *CDKN1B* | 2 | 677 |
| 88 | *KRAS* | 5 | 887 |
| 89 | *MDM2* | 11 | 1,934 |
| 90 | *ATXN2* | 29 | 4,815 |
| 91 | *NOS1* | 29 | 5,556 |
| 92 | *PXN* | 12 | 2,400 |
| 93 | *UBC* | 1 | 2,098 |
| 94 | *ULK1* | 28 | 4,236 |
| 95 | *SMAD9* | 6 | 1,644 |
| 96 | *FOXO1* | 4 | 1,949 |
| 97 | *TBC1D4* | 22 | 4,704 |
| 98 | *SOS2* | 23 | 4,890 |
| 99 | *HIF1A* | 16 | 3,228 |
| 100 | *PGF* | 7 | 793 |
| 101 | *AKT1* | 13 | 1,963 |
| 102 | *RASGRP1* | 17 | 3,073 |
| 103 | *MAP2K1* | 11 | 1,622 |
| 104 | *TSC2* | 42 | 7,137 |
| 105 | *MLST8* | 8 | 1,319 |
| 106 | *PDPK1* | 14 | 2,231 |
| 107 | *ABAT* | 15 | 2,096 |
| 108 | *EEF2K* | 17 | 2,858 |
| 109 | *PRKCB* | 18 | 2,878 |
| 110 | *MAPK3* | 8 | 1,460 |
| 111 | *CDH5* | 12 | 2,760 |
| 112 | *PLCG2* | 32 | 5,034 |
| 113 | *YWHAE* | 6 | 982 |
| 114 | *TP53* | 11 | 1,672 |
| 115 | *FLCN* | 12 | 2,415 |
| 116 | *MAP2K3* | 12 | 1,524 |
| 117 | *NF1* | 59 | 10,808 |
| 118 | *RPS6KB1* | 16 | 2,234 |
| 119 | *PRKCA* | 17 | 2,699 |
| 120 | *MAP2K6* | 12 | 1,485 |
| 121 | *RPTOR* | 34 | 5,368 |
| 122 | *SMAD4* | 11 | 2,071 |
| 123 | *CCBE1* | 11 | 1,661 |
| 124 | *PHLPP1* | 23 | 5,332 |
| 125 | *BCL2* | 4 | 772 |
| 126 | *STK11* | 9 | 1,662 |
| 127 | *MAP2K2* | 11 | 1,630 |
| 128 | *PIK3R2* | 17 | 2,526 |
| 129 | *AKT2* | 13 | 1,966 |
| 130 | *BAX* | 6 | 959 |
| 131 | *AKT1S1* | 6 | 996 |
| 132 | *PRKCG* | 20 | 2,859 |
| 133 | *PLCG1* | 32 | 5,156 |
| 134 | *YWHAB* | 5 | 941 |
| 135 | *ELMO2* | 20 | 2,963 |
| 136 | *SOX18* | 4 | 1,073 |
| 137 | *MYT1* | 23 | 4,033 |
| 138 | *MAPK1* | 8 | 1,361 |
| 139 | *YWHAH* | 2 | 805 |
| 140 | *PRR5* | 9 | 1,599 |
| 141 | *VEGFD* | 7 | 1,326 |
| 142 | *XIAP* | 6 | 1,720 |
| 143 | *GAB3* | 10 | 2,151 |

Additional File 1: Fig. S1. Clinical photos of the patients. (A) Patient 2 (*PIK3CA* mutation) with a long second toe of the left foot. (B) Patient 3 (*PIK3CA* mutation) with left leg overgrowth. (C) Patient 6 (*PIK3CA* mutation) with cutaneous capillary malformation on the neck. (D) Patient 7 (*PIK3CA* mutation) with right leg overgrowth. (E) Patient 8 (*KRAS* mutation) with right leg overgrowth. (F) Patient 11 (*MAP2K3* mutation) with left leg overgrowth. (G) Patient 12 (*GNAQ* mutation) with right ipsilateral overgrowth and nevus flammeus. (H) Patient 13 (*TBC1D4* mutation) with right leg overgrowth. (I) Patient 15 (no mutation identified) with right leg overgrowth and cutaneous capillary malformation.


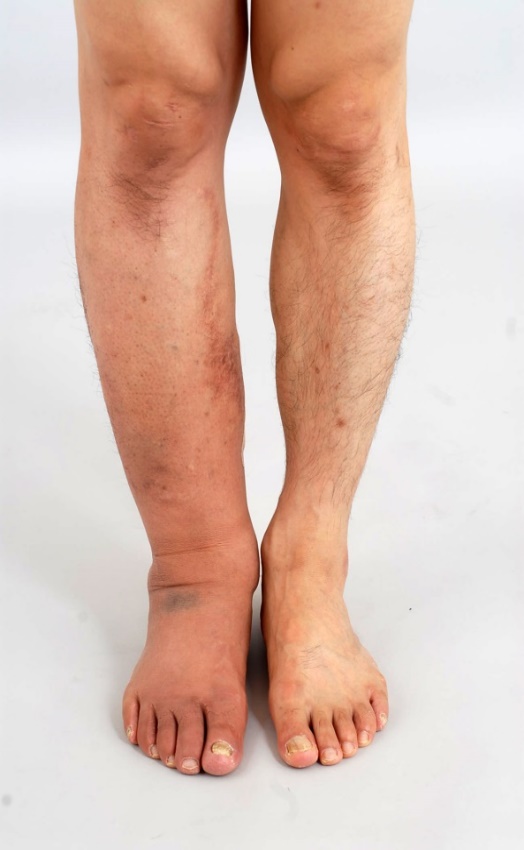

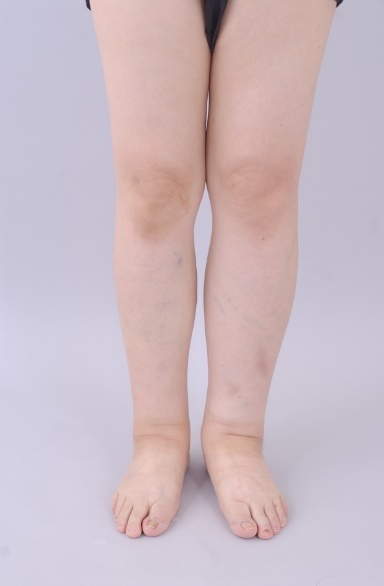


**(B)**


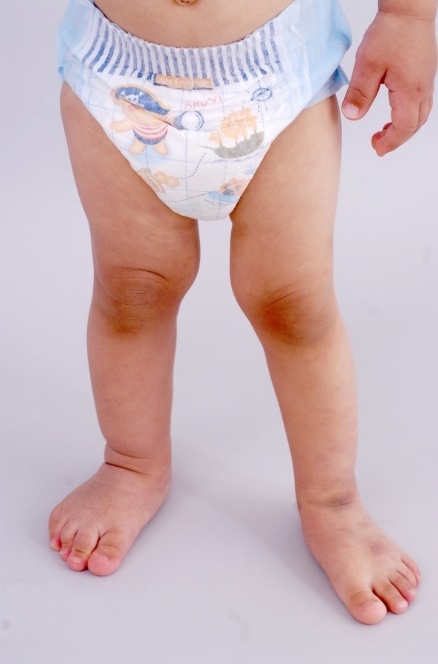


**(D)**


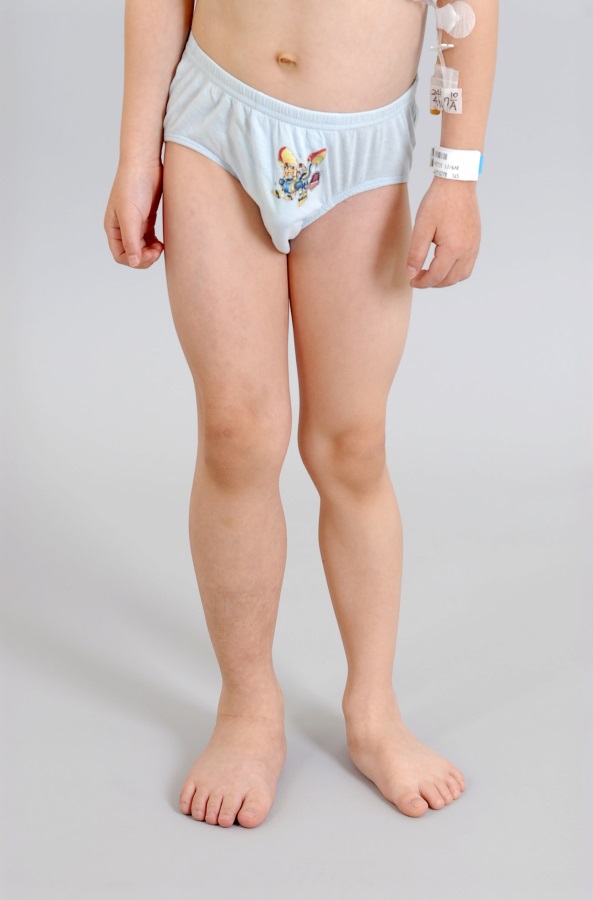


**(E)**


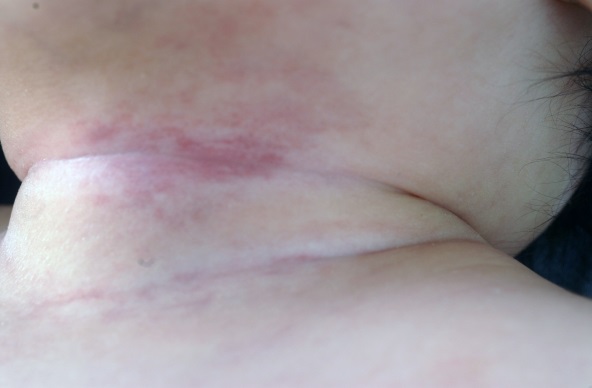


**(C)**


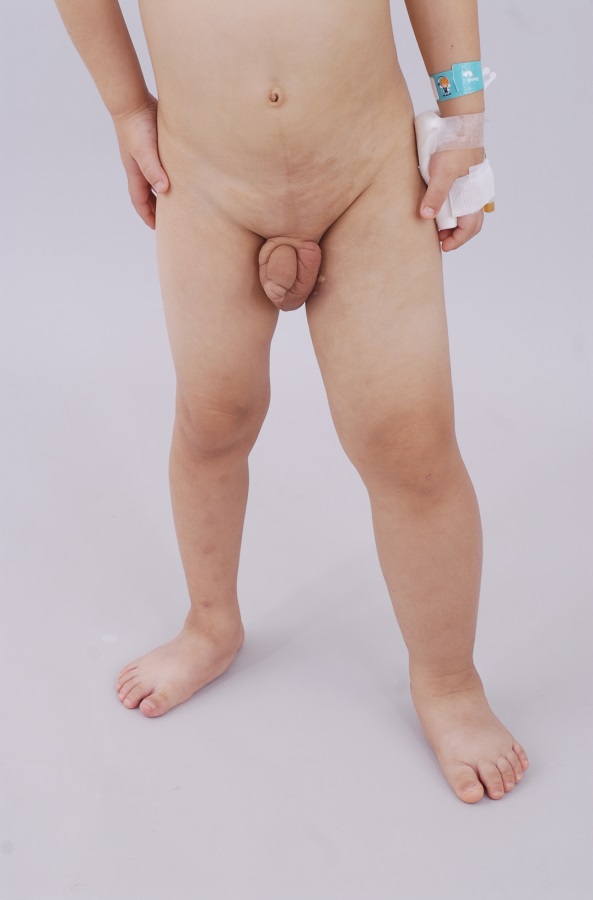


**(F)**


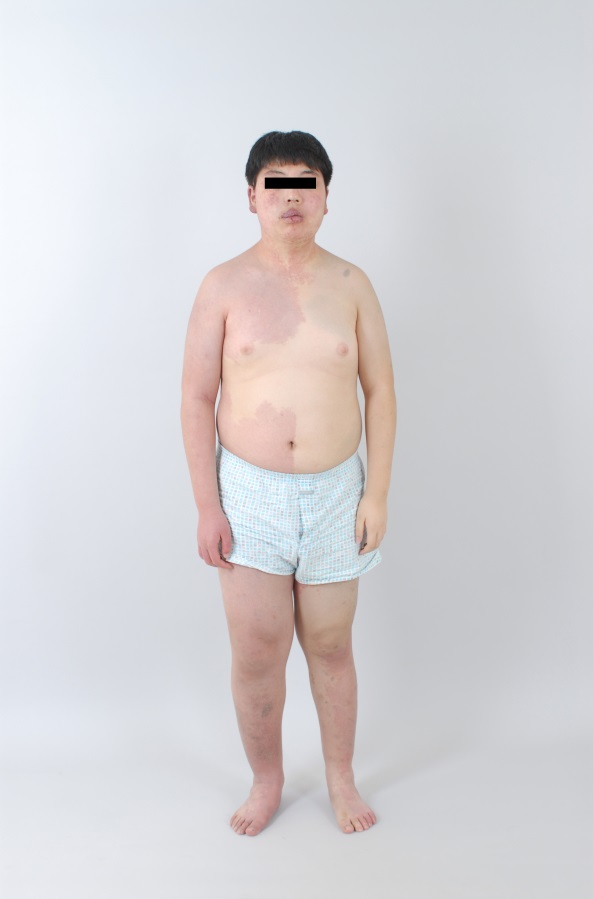


**(H)**


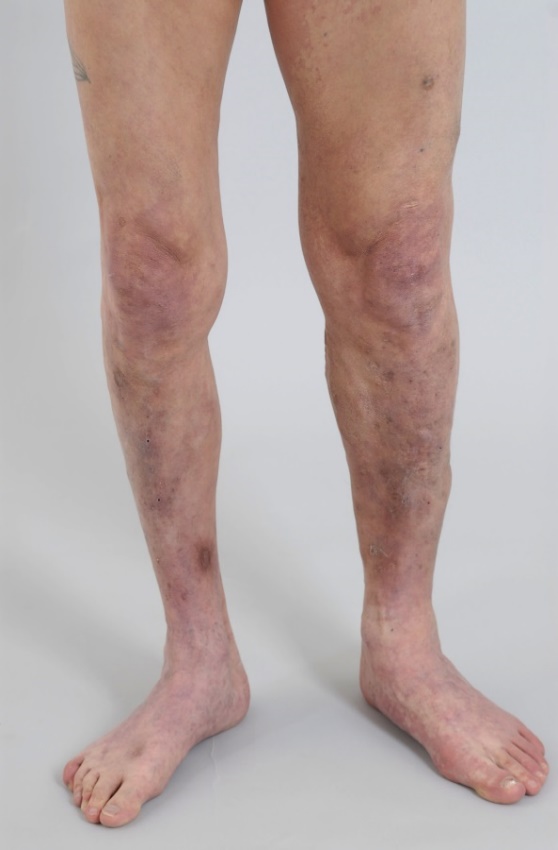


**(I)**


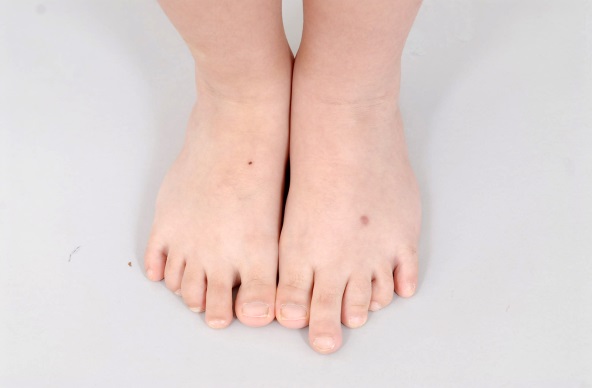


**(A)**

**(G)**
